# Development of ghrelin resistance in a cancer cachexia rat model using human gastric cancer-derived 85As2 cells and the palliative effects of the Kampo medicine rikkunshito on the model

**DOI:** 10.1371/journal.pone.0173113

**Published:** 2017-03-01

**Authors:** Kiyoshi Terawaki, Yohei Kashiwase, Yumi Sawada, Hirofumi Hashimoto, Mitsuhiro Yoshimura, Katsuya Ohbuchi, Yuka Sudo, Masami Suzuki, Kanako Miyano, Seiji Shiraishi, Yoshikazu Higami, Kazuyoshi Yanagihara, Tomohisa Hattori, Yoshio Kase, Yoichi Ueta, Yasuhito Uezono

**Affiliations:** 1 Division of Cancer Pathophysiology, National Cancer Center Research Institute, Chuo-ku, Tokyo, Japan; 2 Tsumura Research Laboratories, Kampo Scientific Strategies Division, Tsumura & Co., Ami-machi, Inashiki-gun, Ibaraki, Japan; 3 Laboratory of Molecular Pathology and Metabolic Disease, Faculty of Pharmaceutical Sciences, Tokyo University of Science, Noda, Chiba, Japan; 4 Department of Physiology, School of Medicine, University of Occupational and Environmental Health, Yahatanishi-ku, Kitakyushu, Fukuoka, Japan; 5 Division of Biomarker Discovery, Exploratory Oncology Research & Clinical Trial Center, National Cancer Center, Kashiwa, Chiba, Japan; 6 Division of Supportive Cancer Research, Exploratory Oncology Research & Clinical Trial Center, National Cancer Center, Chuo-ku, Tokyo, Japan; 7 Innovation Center for Supportive, Palliative and Psychosocial Care, National Cancer Center, Chuo-ku, Tokyo, Japan; Monash University, AUSTRALIA

## Abstract

Cancer cachexia (CC) is a multifactorial disease characterized by decreased food intake and loss of body weight due to reduced musculature with or without loss of fat mass. Patients with gastric cancer have a high incidence of cachexia. We previously established a novel CC rat model induced by human gastric cancer-derived 85As2 cells in order to examine the pathophysiology of CC and identify potential therapeutics. In patients with CC, anorexia is often observed, despite elevation of ghrelin, suggesting that ghrelin resistance may develop in these patients. In this study, we aimed to clarify the occurrence of ghrelin resistance in CC rats accompanied by anorexia and we investigated whether rikkunshito (RKT), a traditional Japanese Kampo medicine that potentiates ghrelin signaling, ameliorated CC-related anorexia through alleviation of ghrelin resistance. 85As2-tumor-bearing rats developed severe CC symptoms, including anorexia and loss of body weight/musculature, with the latter symptoms being greater in cachectic rats than in non-tumor-bearing or pair-fed rats. CC rats showed poor responses to intraperitoneal injection of ghrelin. In CC rats, plasma ghrelin levels were elevated and hypothalamic anorexigenic peptide mRNA levels were decreased, whereas hypothalamic growth hormone secretagogue receptor (*GHS-R*) mRNA was not affected. *In vitro*, RKT directly enhanced ghrelin-induced GHS-R activation. RKT administrated orally for 7 days partly alleviated the poor response to ghrelin and ameliorated anorexia without affecting the elevation of plasma ghrelin levels in CC rats. The expression of hypothalamic orexigenic neuropeptide Y mRNA but not hypothalamic *GHS-R* mRNA was increased by RKT. Thus, the 85As2 cell-induced CC rat model developed ghrelin resistance, possibly contributing to anorexia and body weight loss. The mechanism through which RKT ameliorated anorexia in the CC rat model may involve alleviation of ghrelin resistance by enhancement of ghrelin signaling. These findings suggest that RKT may be a promising agent for the treatment of CC.

## Introduction

Cancer anorexia-cachexia syndrome, which occurs in 80% of patients with advanced cancer, is a multifactorial disease characterized by decreased food intake and loss of body weight and accounts for at least 20% of cancer-related deaths [1–3]. Weight loss, the most prominent clinical feature of cachexia, is observed in 30–80% of patients with cancer, with variations observed according to tumor type [2]. For example, weight loss occurs at a very high frequency (83%) in patients with gastric and pancreatic cancer but is less prominent in patients with breast cancer, acute nonlymphocytic leukemia, and sarcomas [2]. Inflammatory cytokines derived from cancer cells and host immune cells, including interleukin (IL)-1, IL-6, tumor necrosis factor (TNF)-α, and leukemia inhibitory factor (LIF), are involved in the development of cancer anorexia-cachexia [4–7]. However, the mechanisms underlying this syndrome are not fully understood, and appropriate therapies for the treatment of cancer cachexia (CC) have not been established.

In both the diagnosis of and therapy for cancer anorexia-cachexia, management of appetite is important because it reinforces physical strength and improves quality of life (QOL) and maintenance of body weight. Indeed, nutritional intake accounts for 20% of QOL in patients with cancer [8]. Moreover, advanced cancer patients with early satiety have a 30% increased risk of death [9]. Consequently, amelioration of cachexia-related anorexia in patients with cancer is the key to improving both QOL and prognosis.

In a previous study, we established novel cancer anorexia-cachexia rat models by implanting nude rats with MKN45clone85 or 85As2 cells [10], both of which were derived from the human stomach cancer cell line MKN-45 [11]. The model of 85As2-induced CC exhibits earlier and more severe cachexia than the MKN45cl85 model, allowing for more rapid evaluation of CC parameters associated with poor QOL in patients.

Ghrelin, a 28-amino acid peptide, is an endogenous ligand of the growth hormone secretagogue receptor (GHS-R) and is mainly secreted from X/A-like cells in the stomach. Several other tissues, including the hypothalamus and pancreas, express low levels of ghrelin [12, 13]. Ghrelin is the only orexigenic peptide secreted peripherally and is known to have an intense appetite-promoting effect [14]. In addition, ghrelin stimulates growth hormone (GH) secretion, gastric motility, and gastric acid secretion [15]. Moreover, plasma ghrelin increases in response to prolonged fasting and decreases rapidly after feeding owing to its orexigenic effects, suggesting that peripheral ghrelin regulates appetite [16]. In rodents, central or peripheral injection of ghrelin increases food intake and body weight [17]. Therefore, ghrelin may have applications in the therapeutic management of cancer anorexia-cachexia syndrome and has been shown to ameliorate cachexia symptoms in tumor-bearing animal models and patients with cancer [18, 19]. However, in patients with some types of CC, plasma ghrelin levels are elevated, but anorexia still emerges. Therefore, ghrelin resistance may be observed in the pathologic state [20–23], and reduction of ghrelin resistance may be a potential therapeutic target for the treatment of CC.

Rikkunshito (RKT), a traditional Japanese medicine (Kampo medicine), has been approved by the Ministry of Health, Labor, and Welfare of Japan and is widely prescribed as a remedy for various upper gastrointestinal syndromes, such as anorexia, dyspepsia, vomiting, and gastritis. In a multicenter, double-blind, randomized, placebo-controlled study, RKT was shown to ameliorate functional dyspepsia [24]. In various experimental animal models and humans, RKT prevents anorexia through a mechanism involving increased ghrelin secretion [25–28]. Furthermore, RKT has been shown to potentiate ghrelin signaling by enhancing GHS-R activity [29, 30]. We recently demonstrated that RKT ameliorates CC symptoms in a novel 85As2-induced CC rat model [10]. It remains unclear whether enhanced ghrelin secretion or response is related to the specific mechanisms through which RKT prevents cancer anorexia-cachexia in this model.

In this study, we aimed to clarify the occurrence of ghrelin resistance in our anorexia-cachexia rat model and we investigated the sensitivity of these rats to exogenous ghrelin administration. Furthermore, we investigated whether RKT ameliorated anorexia in our CC rat model through a mechanism involving alleviation of ghrelin resistance.

## Materials and methods

### Animal experiment

Six-week-old male F344/NJcl-rnu/rnu rats (CLEA Japan, Tokyo, Japan) were housed individually under a 12:12-h light-dark cycle (lights on at 08:00) at a constant temperature and humidity with *ad libitum* access to food (CE-2; CLEA Japan) and water. Rats were allowed to acclimate to laboratory conditions for 2 weeks prior to experimentation. All studies were performed according to the Guidelines for Animal Experiments and approved by the Committee for Ethics in Animal Experimentation of the National Cancer Center (approval nos. T09-050-M02 and T09-050-C04). These guidelines met the ethical standards concerning experimental animals in Japan, as required by law.

### Cell lines and culture conditions

85As2 cells were established from MKN-45 human gastric cancer cells as described previously [11]. Cells were maintained in RPMI 1640 medium (Nacalai Tesque, Inc., Kyoto, Japan) supplemented with 10% fetal bovine serum (FBS; Invitrogen, Carlsbad, CA, USA), 100 IU/mL penicillin G sodium, and 100 μg/mL streptomycin sulfate under a 5% CO_2_ atmosphere at 37°C. Human embryonic kidney (HEK) 293A cells stably expressing GHS-R were maintained in Dulbecco’s modified Eagle’s medium (DMEM; high-glucose; Wako, Osaka, Japan) supplemented with 10% FBS, 100 IU/mL penicillin G sodium, 100 μg/mL streptomycin sulfate, and 500 μg/mL G418 Disulfate Aqueous Solution (Nacalai Tesque, Inc.) under a 5% CO_2_ atmosphere at 37°C.

### Preparation of the CC model and pair-fed rats

The 85As2 cell-induced CC rat model was prepared as previously described [10]. Briefly, cells were maintained in RPMI 1640 medium supplemented with 10% FBS, 100 IU/mL penicillin G sodium, and 100 μg/mL streptomycin sulfate under a 5% CO_2_ atmosphere at 37°C. 85As2 cells were harvested from subconfluent cultures after brief exposure to 0.25% trypsin and 0.2% ethylenediaminetetraacetic acid. The cells were washed once in serum-free medium and resuspended in phosphate-buffered saline. Rats anesthetized by inhalation of 1–2.5% isoflurane (Mylan, Osaka, Japan) were subcutaneously (s.c.) inoculated with 1 × 10^7^ 85As2 cells/site (tumor-bearing rats) or saline (non-tumor-bearing control rats) in the left and right flanks, respectively, on day 0. The major and minor tumor axes were measured, and the tumor volume was estimated using the following equation: tumor volume (cm^3^) = major axis (cm) × minor axis (cm) × minor axis (cm) × 1/2 [31]. Pair-fed rats received an amount of food that was the same as that consumed by the 85As2-induced CC rats the previous day (days 1–15, the amount of food for pair-fed rats on day 1 was equivalent to that of cachectic rats on day 0). Body weight, organ tissue weights, food and water consumption, plasma glucose level, and orexigenic/anorexigenic peptide hormone mRNA expression in hypothalamic regions were evaluated in each animal. Body weight and food and water consumption were measured daily until day 14 or 15. On day 14 (CC rats and non-tumor-bearing control rats) or 15 (pair-fed rats), the rats were anesthetized by inhalation of 1–2.5% isoflurane, and blood collected from the abdominal aorta was immediately subjected to plasma glucose concentration measurement using a Glucose Pilot System (IWAI Chemicals Company, Tokyo, Japan). The brain was immediately dissected and stored at –80°C until *in situ* hybridization. Organ tissues were immediately dissected and weighed.

### Evaluation of the orexigenic effects of exogenous ghrelin injection

Rats were implanted s.c. with 85As2 cells in both flanks (1 × 10^7^ cells/site) on day 0. Rats inoculated with saline served as the non-tumor-bearing control group. Food and water intake were measured weekly until day 14. After 2 weeks (day 14), the rats were injected intraperitoneally (i.p.) with rat ghrelin (10 nmol/animal; Peptide Institute, Osaka, Japan), and food and water intake were measured for 1 h after injection. The next day, the rats were injected with saline, and food and water intake were measured for 1 h. Saline or ghrelin was injected between 10:00 and 13:00.

### Effects of RKT on attenuation of ghrelin-induced orexigenic effects in CC rats

RKT was manufactured by Tsumura and Co. (Tokyo, Japan) by spray-drying a hot water extract from the following eight crude drugs to form a powdered extract: 18.6% *Atractylodis lanceae rhizoma* (rhizome of *Atractylodes lancea* De Candolle, Compositae), 18.6% *Ginseng radix* (root of *Panax ginseng* C.A. Meyer, Araliaceae), 18.6% *Pinelliae tuber* (tuber of *Pinellia ternate* Breitenbach), 18.6% *Hoelen* (sclerotium of *Poria cocos* Wolf, Polyporaceae), 9.3% *Zizyphi fructus* (fruit of *Zizyphus jujuba* Miller var. *inermis* Rehder, Rhamnaceae), 9.3% *Aurantii nobilis pericarpium* (pericarp of *Citrus unshiu* Markovich, Rutaceae), 4.7% *Glycyrrhizae radix* (root and stolon of *Glycyrrhiza uralensis* Fisher, Leguminosae), and 2.3% *Zingiberis rhizoma* (rhizome of *Zingiber officinale* Roscoe, Zingiberaceae). RKT was dissolved in distilled water (DW) at g/10 mL in our laboratory and administered via oral gavage. Rats were implanted s.c. with 85As2 cells in both flanks (1 × 10^7^ cells/site) on day 0. Rats inoculated with saline served as the non-tumor-bearing control group. Tumor-bearing rats were divided into two groups: a treatment (85As2 + RKT) group and a tumor-bearing control (85As2 + DW) group. The treatment group was orally administered RKT twice daily at 1 g/kg/day for 7 days (from day 14 to day 20). The tumor-bearing control group and non-tumor-bearing rats were administered DW (10 mL/kg) over the same period. For the evaluation of orexigenic effects induced by ghrelin, rats were injected i.p. with ghrelin (10 nmol/animal) on day 21, and food intake was measured for 1 h or 22 h after injection. The next day, rats were injected i.p. with vehicle (saline) as a control, and food intake was measured for 1 h or 22 h.

### Effects of RKT on anorexia-cachexia, plasma ghrelin, and hypothalamic orexigenic/anorexigenic peptide in the CC model

In a procedure similar to that used for evaluation of the effects of RKT on attenuation of ghrelin-induced orexigenic effects in the 85As2-induced CC rats, three groups (control + DW, 85As2 + DW, and 85As2 + RKT), were prepared. Food intake and body weight were measured weekly until day 14 and daily thereafter. In addition to body weight gain and loss after administration, body weight data on days 14–21 after RKT or DW administration were expressed using the following relationship as daily comparisons: % of pre (%) = body weight of each animal after administration (on days 15–21)/body weight of each animal before administration (on day 14) × 100. Food intake after RKT or DW administration was calculated as the daily average value from days 16 to 21 as follows: food intake gain (g) = daily average food intake of each animal (after)–food intake of each animal (before).

On day 21, for the measurement of plasma acyl ghrelin levels, the rats were anesthetized with isoflurane, blood collected from the abdominal aorta was centrifuged (1700 × *g* for 10 min), and the plasma was stored at –80°C until analysis. For measurement of hypothalamic neuropeptide Y (*NPY*) and *GHS-R* mRNAs and *in situ* hybridization, the brain or hypothalamus was immediately dissected and stored at –80°C until analysis.

### Measurement of plasma ghrelin concentrations

Blood collected from the abdominal aorta was centrifuged (1700 × *g* for 10 min). To prevent degradation of acyl ghrelin, the plasma was immediately treated with 1/10 volume of 1 M HCl. The plasma was stored at –80°C until analysis. Plasma acyl ghrelin levels were measured using an enzyme-linked immunosorbent assay (ELISA) kit (SCETI, Tokyo, Japan) according to the manufacturer’s instructions.

### RNA extraction, reverse transcription, and real-time Polymerase Chain Reaction (PCR)

Real-time PCR was performed as previously described [32]. Briefly, the hypothalamic area was dissected on an ice-cold metal plate, and total RNA was isolated using an Isogen kit (Nippon Gene Co., Ltd., Tokyo, Japan) according to the manufacturer’s instructions. First-strand cDNA was reverse-transcribed from 5 μg total RNA using the SuperScript First-Strand Synthesis System (Invitrogen) in a final volume of 100 μL. Diluted cDNA (2 μL) was amplified in a rapid thermal cycler (LightCycler; Roche Diagnostics, Barcelona, Spain) using LightCycler 480 SYBR Green I MasterMix (Roche) and the following primers: *GHS-R1a*, (forward) 5ʹ-GAAAGCAAACACCACCACAG-3ʹ and (reverse) 5ʹ-AGGAAGCTATGGCGGAGAC-3ʹ; *NPY*, (forward) 5ʹ- CCGCTCTGCGACACTACAT-3ʹ and (reverse) 5ʹ-TGTCTCAGGGCTGGATCTCT-3ʹ; GAPDH, (forward) 5ʹ-CCCCCAATGTATCCGTTGTG-3ʹ and (reverse) 5ʹ-TAGCCCAGGATGCCCTTTAGT-3ʹ. PCR products were quantified using LightCycler 480 software. The amount of target mRNA in the experimental group relative to that in the control group was determined from the resulting fluorescence and threshold values (CT) using the 2^-ΔΔCT^ method [33].

### *In situ* hybridization

*In situ* hybridization was performed as previously described [10, 34]. Briefly, frozen 12-μm-thick coronal brain sections were prepared in a cryostat at –20°C, thawed, and mounted onto gelatin/chrome alum-coated slides. The paraventricular nucleus (PVN), arcuate nucleus (ARC), and lateral hypothalamic area (LHA) were identified according to the Paxinos and Watson atlas [35] and confirmed by microscopy. Hybridization was conducted under a Nescofilm coverslip (Bando Chemical IMD, Osaka, Japan). [^35^S]3ʹ-end-labeled deoxyoligonucleotides complementary to transcripts encoding NPY (5ʹ-GGAGTAGTATCTGGCCATGTCCTCTGCTGGCGCGTC-3ʹ), agouti-related protein (AgRP; 5ʹ-CGACGCGGAGAACGAGACTCGCGGTTCTGTGGATCTAGCACCTCTGCC-3ʹ), proopiomelanocortin (POMC; 5ʹ-CTTCTTGCCCAGCGGCTTGCCCCAGCAGAAGTGCTCCATGGACTAGGA-3ʹ), cocaine- and amphetamine-regulated transcript (CART; 5ʹ-TGGGGACTTGGCCGTACTTCTTCTCATAGATCGGAATGC-3ʹ), orexin (5ʹ-TTCGTAGAGACGGCAGGAACACGTCTTCTGGCGACA-3ʹ), corticotropin-releasing hormone (*CRH*; 5ʹ-CAGTTTCCTGTTGCTGTGAGCTTGCTGAGCTAACTGCTCTGCCCTGGC-3ʹ), thyrotropin-releasing hormone (*TRH*; 5ʹ-GTCTTTTTCCTCCTCCTCCCTTTTGCCTGGATGCTGCGCTTTTGTGAT-3ʹ), and melanin-concentrating hormone (*MCH*; 5ʹ-CCAACAGGGTCGGTAGACTCGTCCCAGCAT-3ʹ) were used as gene-specific probes.

Total counts of 6 × 10^5^ cpm/slide for NPY, AgPR, POMC, CART, MCH, TRH, and CRH and 4 × 10^5^ cpm/slide for orexin were used. Hybridized sections containing the ARC, LHA, and PVN regions were exposed to autoradiography film (Hyperfilm; Amersham, Buckinghamshire, UK) for 4 days for orexin and 7 days for NYP, AgRP, POMC, CART, MCH, and CRH. Autoradiographic images were captured at 40× magnification and quantified using an MCID imaging analyzer (Imaging Research, St. Catherines, ON, Canada). Mean absorbance was measured and compared with simultaneously exposed ^14^C microscale samples (Amersham). The standard curve was fitted according to the absorbance of the ^14^C microscale on the same film.

### Evaluation of ghrelin signaling using the CellKey system

Measurement of ghrelin signaling by the CellKey system (MDS Sciex, Ontario, Canada) was performed using previously described methods [36]. Briefly, a 96-well CellKey plate was washed with sterile water 1 h after coating the surface with poly-d-lysine. HEK293A cells expressing GHS-R were seeded at a density of 25,000 cells/well and incubated under a 5% CO_2_ atmosphere at 37°C. The cells were treated with vehicle or ghrelin at 28°C. Ghrelin was prepared at 10× final concentration (10^−10^, 3 × 10^−10^, and 10^−9^ M) diluted with Hank’s balanced salt solution (1.3 mM CaCl_2_∙2H_2_O, 0.81 mM MgSO_4_, 5.4 mM KCl, 0.44 mM KH_2_PO_4_, 4.2 mM NaHCO_3_, 136.9 mM NaCl, 0.34 mM Na_2_HPO_4_, and 5.6 mM d-glucose) containing 20 mM 4-(2-hydroxyethyl)-1-piperazineethanesulfonic acid (HEPES). Cell plates were washed three times with pH 7.4 Hank’s balanced salt solution containing 20 mM HEPES (HHBS), and 135 μL HHBS was added to each well. Plates were allowed to equilibrate at 28°C for 30 min. A baseline was established for 5 min, 15 μL of ghrelin solution or vehicle (HHBS) was added, and the impedance between the electrodes was measured every 10 s over 15 min to measure ghrelin signaling. To evaluate the effects of RKT on ghrelin signaling activation, 135 μL RKT solution (10 μg/mL) was added to the plates after the final wash and allowed to equilibrate at 28°C for 1 h in the CellKey device. The impedance was then measured after addition of ghrelin solution or vehicle.

### Effects of long-term feeding of an RKT-containing diet on anorexia in the CC model

Rats were divided into three groups: a non-tumor-bearing (control + CE-2) group, a tumor-bearing control (85As2 + CE-2) group, and a treatment (85As2 + RKT-1% diet) group. Rats were implanted s.c. with 85As2 cells in both flanks (1 × 10^6^ cells/site) on day 0 as the tumor-bearing rats. Rats inoculated with saline served as the non-tumor-bearing control group. The treatment group was fed an RKT-1% diet (CE-2 diet containing 1% RKT) with *ad libitum* access during the experiment (from day –7 to day 28). The tumor-bearing control and non-tumor-bearing group were fed CE-2 over the same period. Tumor volume, average daily food intake, and body weight were measured weekly over this period.

### Statistical analyses

All data are expressed as the mean ± standard error of the mean (SEM). Differences between groups were evaluated using Aspin–Welch’s *t*-tests, paired *t*-tests, one-way analysis of variance (ANOVA) followed by Dunnett’s multiple comparison tests, one-way ANOVA followed by post-hoc Bonferroni’s multiple comparison tests, or two-way repeated-measures ANOVA followed by post-hoc Bonferroni tests. The significance level α was set at 0.05, and differences with a two-sided *p-*value of less than 0.05 were considered significant. All statistical analyses were performed using GraphPad Prism version 5 (GraphPad Software Inc., San Diego, CA, USA).

## Results

### Characterization of the 85As2-induced CC rat model compared with pair-fed rats

**Fig 1:**
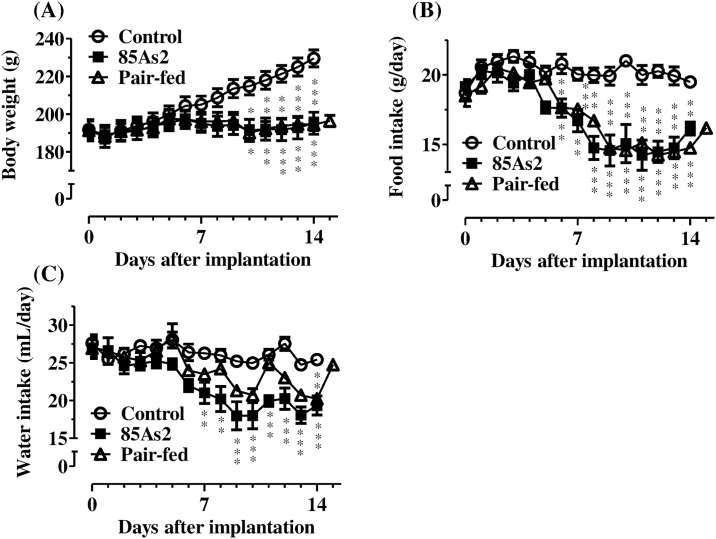
Comparison of pair-fed and 85As2-induced CC rats. Changes in (A) body weight, (B) food intake, and (C) water intake. Rats were inoculated subcutaneously with 85As2 cells in both flanks (1 × 10^7^ cells/site) on day 0. Rats inoculated with saline served as a non-tumor-bearing control group. The pair-fed rats received an amount of food that was the same as that consumed by the 85As2-induced CC rats for the previous day (days 1–15). Each data point represents the mean ± SEM of six rats. Differences between groups were evaluated using two-way repeated measures ANOVA followed by post-hoc Bonferroni tests; *p < 0.05, **p < 0.01, ***p < 0.001 versus the corresponding control group.

Body weight and food and water intake were examined in rats until 14 days after treatment with 85As2 (or vehicle) or the beginning of pair-feeding. Subcutaneous implantation of 85As2 cells induced progressive tumor growth in the rats; tumor volumes and weights on 14 day after implantation of 85As2 cells are shown in Table 1. The body weights of non-tumor-bearing control rats continued to increase during the experiment, whereas the body weights of rats in the 85As2 and pair-fed groups did not. Two-way repeated measures ANOVA of body weights in all groups revealed significant effects of time (day) [F (14, 210) = 34.98, *p* < 0.0001] and treatment (group) × time interaction [F (28, 210) = 22.59, *p* < 0.0001], but not treatment [F (2, 210) = 2.789, not significant (n.s.)]. The body weights of rats in the 85As2 group on day 10 were significantly lower than those in the control group, and thereafter, the differences gradually increased (two-way repeated measures ANOVA followed by Bonferroni post-hoc tests, day 10: *p* < 0.05; day 11: *p* < 0.01; days 12–14: *p* < 0.001; Fig 1A). On the other hand, pair-fed rats showed changes in body weight over time similar to those in 85As2-induced CC rats, and their body weight was significantly lower than that in the control group (two-way repeated measures ANOVA followed by post-hoc Bonferroni tests, day 10: *p* < 0.05; days 11–12: *p* < 0.01; days 13–14: *p* < 0.001; Fig 1A). Reduced food intake was observed in the 85As2 group as compared to the control group; therefore, food intake in the pair-fed group was reduced similar to that in the 85As2 group. Two-way repeated measures ANOVA of food intake in all groups revealed significant effects of treatment [F (2, 210) = 35.05, *p* < 0.0001], time [F (14, 210) = 31.50, *p* < 0.0001], and treatment × time interaction [F (28, 210) = 7.26, *p* < 0.0001]. Reduced food intake in the 85As2 group as compared to control animals was observed as of 6 day after implantation, and the difference became greater 8 days later. Thereafter, the reduction was stable and persisted (two-way repeated measures ANOVA followed by Bonferroni post-hoc tests, days 6–7: *p* < 0.01; days 8–14: *p* < 0.001; Fig 1B). Similar to the observation in 85As2-induced CC rats, food intake in pair-fed rats was significantly reduced (two-way repeated measures ANOVA followed by Bonferroni post-hoc tests, days 6, 8, and 14: *p* < 0.01; days 7 and 9–13: *p* < 0.001; Fig 1B). Additionally, muscle (greater pectoral, gastrocnemius, and tibialis anterior), adipose tissue (epididymal and mesentery fat), liver, spleen, and kidney weights were substantially decreased in both pair-fed and 85As2-induced CC rats as compared to control rats (Table 1). Plasma glucose levels also were significantly lower in both pair-fed and CC rats than in the control rats. Body weight, after subtraction of tumor weight, was lower in the cachectic than in the pair-fed rats. Furthermore, greater pectoral muscle weight in the 85As2 group was significantly lower than that in in the pair-fed group. Water intake was reduced in the 85As2 group as compared to that in the control group, and slightly reduced in the pair-fed group. Two-way repeated measures ANOVA of water intake in all groups revealed significant effects of treatment [F (2, 210) = 8.744, *p* < 0.01], time [F (14, 210) = 26.17, *p* < 0.0001], and treatment × time interaction [F (28, 210) = 4.318, *p* < 0.0001]. The reduction in the CC rats was significant, stable, and persistent (two-way repeated measures ANOVA followed by Bonferroni post-hoc tests, days 7–8: *p* < 0.01; days 9–14: *p* < 0.001; Fig 1C). However, unlike CC rats, water intake in pair-fed rats showed a significant reduction only on day 14.

**Table 1:** Body, tumor, muscle, fat, organ weights and plasma glucose levels in pair-fed and 85As2-induced CC rats.

		Control	85As2	Pair-fed
Tumor weight (T.W.)		0.00 ± 0.00	2.46 ± 0.42	0.00 ± 0.00
Tumor volume		0.00 ± 0.00	7.71 ± 1.51	0.00 ± 0.00
Body weight (B.W.)		229.53 ± 4.47	195.13 ± 5.83**	196.36 ± 2.99***
B.W.–T.W.		229.53 ± 4.47	192.67 ± 6.02***	196.36 ± 2.99***
B.W. (pre-inoculation)		191.70 ± 5.47	191.63 ± 4.67	191.32 ± 5.56
% Change (post/pre)		119.93 ± 1.84	100.62 ± 2.87***	101.93 ± 1.89***
Muscle weights				
	Greater pectoral	2.22 ± 0.13	1.80 ± 0.03*	1.93 ± 0.04#
	Gastrocnemius	1.24 ± 0.03	1.06 ± 0.03**	1.08 ± 0.02***
	Tibialis anterior	0.46 ± 0.01	0.39 ± 0.02**	0.40 ± 0.01**
	Soleus	0.07 ± 0.01	0.07 ± 0.00	0.07 ± 0.00
	Total muscle	5.76 ± 0.19	4.84 ± 0.12**	5.03 ± 0.09*
Fat weights				
	Epididymis	2.31 ± 0.19	1.70 ± 0.08*	1.80 ± 0.11*
	Perirenal	1.63 ± 0.25	0.98 ± 0.10	1.05 ± 0.19
	Mesentery	1.52 ± 0.16	0.76 ± 0.03**	0.88 ± 0.15*
	Total fat	5.46 ± 0.56	3.45 ± 0.20*	3.73 ± 0.44*
Organ weights				
	Liver	10.15 ± 0.23	7.73 ± 0.30***	7.72 ± 0.15***
	Spleen	0.57 ± 0.02	0.46 ± 0.03*	0.48 ± 0.02*
	Kidney	2.25 ± 0.12	1.91 ± 0.07*	1.92 ± 0.03*
Plasma glucose		142.67 ± 2.40	131.50 ± 1.93***	130.83 ± 3.56**

Rats were s.c. inoculated with 85As2 cells (1 × 10^7^ cells/site) or injected with saline in both flanks. Data are expressed as the mean ± SEM of six rats. All weight data are expressed in grams. Plasma glucose data are expressed in mg/dL. Tumor volume was estimated using the following equation: tumor volume (cm^3^) = major axis (cm) × minor axis (cm) × minor axis (cm) × 1/2. Tumor weight and volume are expressed as the total for both sites. Body and tissue weights were measured on day 14 (85As2 and control groups) or 15 (pair-fed group). % Change (post/pre) was determined as follows: % change = tumor-free body weight (B.W.–T.W.) on day 14 or 15/body weight of pre-inoculation with 85As2 cells or saline on day 0 × 100. Values for bilateral muscle tissues represent the mean of those for the 2 unilateral tissues. Differences between groups were evaluated using the Aspin–Welch *t*-test.

*p < 0.05,

**p < 0.01,

***p < 0.001 vs. the control group,

^#^ *p* < 0.05 vs. the 85As2 group.

**Fig 2:**
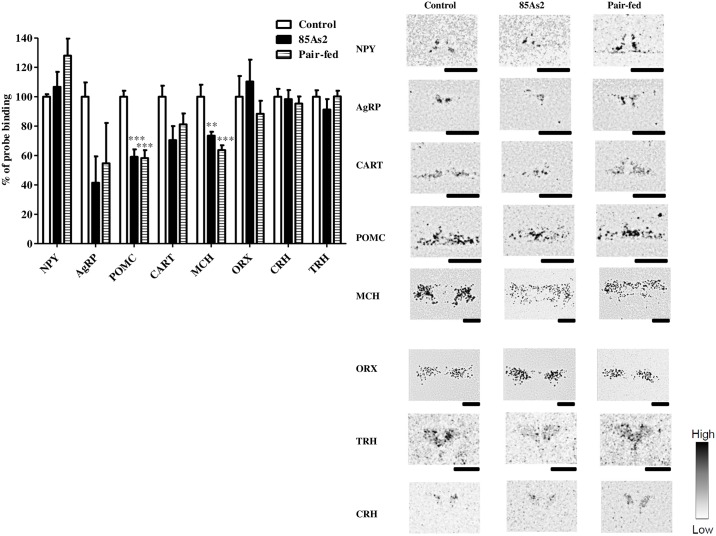
Expression of mRNAs encoding neuropeptide Y (*NPY*), agouti-related protein (*AgRP*), proopiomelanocortin (*POMC*), and cocaine- and amphetamine-regulated transcript (*CART*) in the arcuate nucleus (ARC); corticotropin-releasing hormone (*CRH*) and thyrotropin-releasing hormone (*TRH*) in the paraventricular nucleus (PVN); and orexin (*ORX*) and melanin-concentrating hormone (*MCH*) in the lateral hypothalamic area (LHA) in control, pair-fed, and 85As2-induced CC rats 2 weeks after implantation. Rats were inoculated subcutaneously with 85As2 cells in both flanks (1 × 10^7^ cells/site) on day 0. Rats inoculated with saline served as a non-tumor-bearing control group. The pair-fed rats received an amount of food that was the same as that consumed by the 85As2-induced CC rats the previous day (days 1–15). *In situ* hybridization was performed 2 weeks after implantation (pair-fed rats: day 15). Representative autoradiographs of sections hybridized with a ^35^S-labeled oligodeoxynucleotide probe complementary to the relevant mRNA are shown. The signal intensity ranged from high (black) to low (white). Black bar = 1 mm. Each column represents the mean ± SEM of six rats. Differences between groups were evaluated using one-way ANOVA followed by post-hoc Bonferroni tests; **p < 0.01, ***p < 0.001 versus the corresponding control group.

mRNA levels of hypothalamic feeding-regulated peptide were evaluated 14 days after implantation of 85As2 cells. One-way ANOVA of *POMC* and *MCH* mRNA expression in all groups revealed significant effects [*POMC*, F (2, 15) = 23.98, *p* < 0.0001; *MCH*, F (2, 15) = 12.42, *p* < 0.001]. The mRNA levels of *POMC* in the ARC and *MCH* in the LHA were significantly lower in 85As2-induced CC rats than in control rats (one-way ANOVA followed by post-hoc Bonferroni’s multiple comparison tests, *p* < 0.001, *p* < 0.01, respectively; Fig 2). One-way ANOVA of *NPY*, *AgRP*, *CART*, *ORX*, *CRH*, and *TRH* mRNA expression in all groups revealed no significant effects [*NPY*, F (2, 15) = 2.611, *n*.*s*.; *AgRP*, F (2, 15) = 2.349, *n*.*s*.; *CART*, F (2, 15) = 3.385, *n*.*s*.; *ORX*, F (2, 15) = 0.7201, *n*.*s*.; *CRH*, F (2, 15) = 0.1887, *n*.*s*.; *TRH*, F (2, 15) = 0.9264, *n*.*s*.]. mRNA levels of *NPY*, *AgRP*, and *CART* in the ARC, *ORX* in the LHA, and *CRH* and *TRH* in the PVN were not different from those in control rats. On the other hand, in pair-fed rats, expression levels of all peptides exhibited variations similar to those in CC rats (*POMC*, *p* < 0.001; *MCH*, *p* < 0.001, *NPY*, *n*.*s*.; *AgRP*, *n*.*s*.; *CART*, *n*.*s*.; *ORX*, *n*.*s*.; *CRH*, *n*.*s*.; *TRH*, *n*.*s*., one-way ANOVA followed by post-hoc Bonferroni’s multiple comparison tests; Fig 2).

### Attenuation of orexigenic effects induced by ghrelin in 85As2-induced CC rats

**Fig 3:**
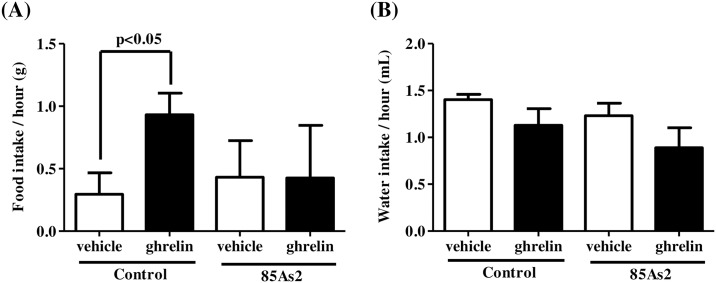
Attenuation of orexigenic effects induced by ghrelin in 85As2-induced CC rats. (A) Food intake and (B) water intake following i.p. injection of ghrelin or vehicle. Rats were inoculated subcutaneously with 85As2 cells in both flanks (1 × 10^7^ cells/site) on day 0. Rats inoculated with saline served as a non-tumor-bearing control group. Rats were injected i.p. with 10 nmol ghrelin, and 1-h food and water intake were measured on day 14. Each column represents the mean ± SEM of five rats. Differences between vehicle and ghrelin in each group were evaluated using paired *t*-tests.

Ghrelin-induced orexigenic effects were examined in rats 2 weeks (day 14) after treatment with 85As2 or vehicle. Tumor growth (4.32 ± 0.45 cm^3^) and cachexia symptoms, such as body weight loss and anorexia, were induced in rats implanted with 85As2 cells (body weight: control group, 238.90 ± 2.85 g, 85As2 group, 188.68 ± 3.39 g, *p* < 0.001; daily food intake: control group, 22.79 ± 0.42 g, 85As2 group, 15.31 ± 1.32 g, *p* < 0.001; daily water intake: control group, 27.99 ± 0.84 mL, 85As2 group, 18.70 ± 0.48 mL, *p* < 0.001; two-way repeated measures ANOVA followed by Bonferroni post-hoc tests), which is consistent with our previous [10] and present (Fig 1) findings. In non-tumor-bearing control rats, i.p. injection of ghrelin induced a significant increase in 1-h food intake as compared to vehicle (saline) injection (paired *t*-tests, *p* < 0.05). However, in 85As2-bearing CC rats, i.p. injection of ghrelin did not change 1-h food intake as compared to vehicle injection (Fig 3A). In contrast, there were no differences in 1-h water intake following i.p. injection of ghrelin between groups (Fig 3B). On the other hand, two-way repeated measures ANOVA of 1-h food intake revealed no significant effects of treatment (group) [F (1, 8) = 0.4732, *n*.*s*.], injection [F (1, 8) = 1.121, *n*.*s*.] and treatment × injection interaction [F (1, 8) = 1.425, *n*.*s*.]. Additionally, two-way repeated measures ANOVA of 1-h water intake revealed no significant effects of treatment (group) [F (1, 8) = 4.468, *n*.*s*.], injection [F (1, 8) = 1.518, *n*.*s*.] and treatment × injection interaction [F (1, 8) = 0.05516, *n*.*s*.].

### Enhancement of ghrelin signaling by RKT in HEK293A cells expressing GHS-R

**Fig 4:**
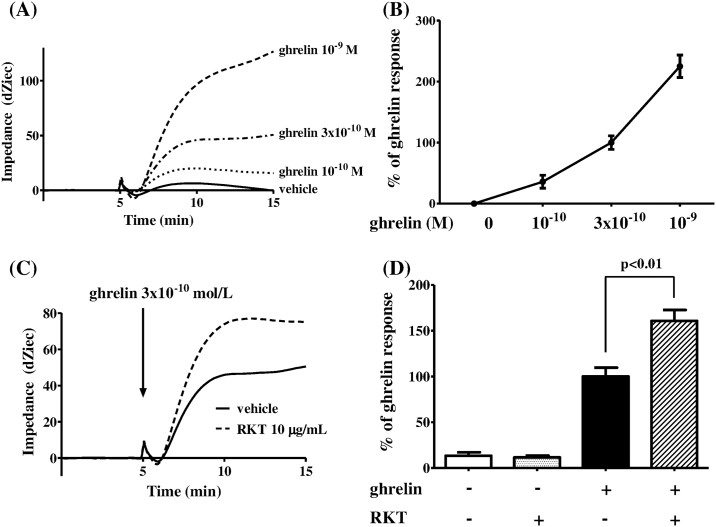
Enhancement of ghrelin signaling by RKT in HEK293A cells expressing GHS-R. (A) Time-dependent changes in impedance induced by stimulation with ghrelin at different concentrations (10^−10^, 3 × 10^−10^, or 10^−9^ M) in HEK293A cells expressing GHS-R. (B) Response to 3 × 10^−10^ M ghrelin at 10 min after injection. (C) Time-dependent changes in response to 3 × 10^−10^ M ghrelin at 60 min after pretreatment with 10 μg/mL RKT. (D) Responses to 3 × 10^−10^ M ghrelin at 10 min after injection. Each column represents the mean ± SEM of six determinations. Differences between groups were evaluated using two-way repeated measures ANOVA followed by Bonferroni post-hoc tests. RKT: rikkunshito; GHS-R: growth hormone secretagogue receptor.

Ghrelin stimulation in HEK293A cells expressing GHS-R caused Gq protein-specific impedance changes in a concentration-dependent manner (Fig 4A and 4B). The impedance changes induced by 3 × 10^−10^ M ghrelin were enhanced by pretreatment with 10 μg/mL RKT (Fig 4C); RKT enhanced the ghrelin signaling response up to 160% ± 11.9% (Fig 4D). Two-way repeated measures ANOVA of the ghrelin signaling response revealed significant effects of drug (with or without RKT) [F (1, 10) = 13.06, *p* < 0.01], treatment (with or without ghrelin) [F (1, 10) = 235.1, *p* < 0.0001], and drug × treatment interaction [F (1, 10) = 16.57, *p* < 0.01]. The enhancement of the ghrelin signaling response by RKT was significant (two-way repeated measures ANOVA followed by Bonferroni post-hoc tests, *p* < 0.01; Fig 4D).

### Alleviation of ghrelin resistance in 85As2-induced CC rats by RKT

**Fig 5:**
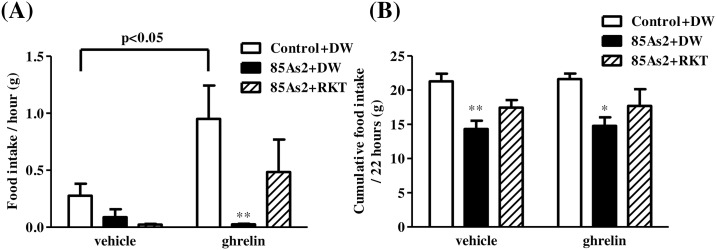
Effects of RKT on attenuation of ghrelin-induced orexigenic effects in 85As2-induced CC rats. (A) Effects of RKT on attenuated ghrelin-induced orexigenic effects (increase in 1-h food intake) in 85As2-induced CC rats. (B) Cumulative food intake (22 h) following vehicle or ghrelin injection in all groups. Rats were implanted s.c. with 85As2 cells in both flanks (1 × 10^7^ cells/site). Two weeks after implantation, RKT (1 g/kg/day) or DW was administered orally twice a day for 7 days. Rats inoculated with saline served as a non-tumor-bearing control group and were administered DW. Rats were injected i.p. with ghrelin (10 nmol) after administration of RKT or DW for 7 days, and 1-h food intake and 22-h cumulative food intake were measured. The next day, all rats were injected i.p. with vehicle (saline) as a control, and 1-h and 22-h food intake was measured. Each data column represents the mean ± SEM of 5–6 rats. Differences between vehicle and ghrelin treatments in each group were evaluated using paired *t*-tests. Differences between groups were evaluated using two-way repeated measures ANOVA followed by Bonferroni post-hoc tests; *p < 0.05, **p < 0.01 versus the Control + DW-treated group. RKT: rikkunshito; DW: distilled water.

In 85As2-implanted rats, cachexia symptoms, such as body weight loss and anorexia, clearly developed at day 14 after implantation (body weight: Control + DW group, 227.7 ± 5.1 g; 85As2 + DW group, 186.7 ± 6.7 g; 85As2 + RKT group, 189.6 ± 7.8 g; *p* < 0.001, *p* < 0.01 versus Control + DW, respectively; and daily food intake: Control + DW group, 22.12 ± 0.58 g; 85As2 + DW group, 14.91 ± 1.36 g; 85As2 + RKT group, 14.24 ± 1.04 g; *p* < 0.001, *p* < 0.001 versus Control + DW, respectively) consistent with our other findings (Figs 1 and 3). Thereafter, the rats were administered RKT or DW for 7 days. In non-tumor-bearing rats (Control + DW), i.p. injection of ghrelin induced a significant increase in 1-h food intake compared to vehicle injection (paired t-tests, *p* < 0.05; Fig 5A). In contrast, in 85As2-bearing CC rats (85As2 + DW), i.p. injection of ghrelin hardly increased the 1-h food intake as compared to vehicle injection. Two-way repeated measures ANOVA of 1-h food intake in all groups revealed significant effects of i.p. injection [F (1, 14) = 5.502, *p* < 0.05] and treatment (group) [F (2, 14) = 4.606, *p* < 0.05], but not i.p. injection × treatment interaction [F (2, 14) = 1.953, n.s.]. The difference between the Control + DW + ghrelin group and the 85As2 + DW + ghrelin group was significant (two-way repeated measures ANOVA followed by Bonferroni post-hoc tests, *p* < 0.01; Fig 5A). RKT partly, but not significantly, blocked the decrease in ghrelin-induced 1-h food intake in 85As2-bearing CC rats (Fig 5A). There were no significant differences between the 85As2 + RKT group and non-tumor-bearing rats (Control + DW). Thereafter, food intake following ghrelin injection was measured up to 22 h. Unlike 1-h food intake, there were no differences between vehicle and ghrelin in all groups (paired *t*-tests, n.s.), showing that the effects of ghrelin injection quickly disappeared (in 1–2 h from the preliminary results). Two-way repeated measures ANOVA of cumulative 22-h food intake in all groups revealed a significant effect of treatment (group) [F (2, 14) = 7.280, *p* < 0.01], but not i.p. injection [F (1, 14) = 0.1694, n.s.] and i.p. injection × treatment interaction [F (2, 14) = 0.005446, n.s.]. In 85As2-bearing CC rats (85As2 + DW), 22-h food intake following i.p. injection of both vehicle and ghrelin was significantly decreased compared to that in non-tumor-bearing rats (22-h food intake following vehicle injection: Control + DW, 21.28 ± 1.13 g; 85As2 + DW, 14.33 ± 1.19 g; 22-h food intake following ghrelin injection: Control + DW, 21.61 ± 0.82 g; 85As2 + DW, 14.78 ± 1.25 g, two-way repeated measures ANOVA followed by Bonferroni post-hoc tests, *p* < 0.01, *p* < 0.05, respectively; Fig 5B). RKT partly, but not significantly, alleviated the decrease in 22-h food intake in 85As2-bearing CC rats (85As2 + RKT, 17.45 ± 1.10 g, 17.69 ± 2.44 g, vehicle and ghrelin, respectively; Fig 5B).

### Effects of RKT on anorexia-cachexia symptoms, plasma ghrelin levels, and hypothalamic *GHS-R* and *NPY* gene expression in 85As2-induced CC rats

**Fig 6:**
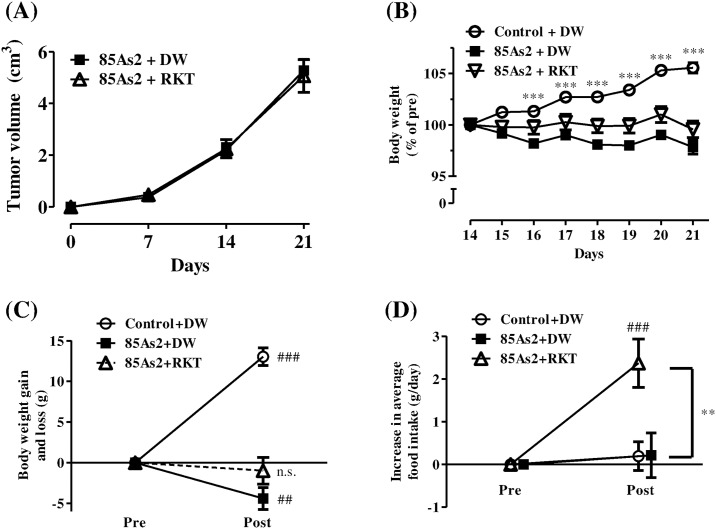
Effects of RKT on tumor growth, body weight loss, and anorexia in the 85As2-induced CC model. Changes in (A) tumor volume over time and (B) body weight after RKT or DW administration. Comparison of (C) body weight gain and (D) increase in average daily food intake before and after RKT or DW administration in individual rats. Rats were implanted subcutaneously with 85As2 cells in both flanks (1 × 10^7^ cells/site) on day 0. RKT (1 g/kg/day) or DW was administered orally twice a day for 7 days from day 14. Rats inoculated with saline served as a non-tumor-bearing control group and were administered DW. Each data point represents the mean ± SEM of 14–16 rats. Differences between groups were evaluated using two-way repeated measures ANOVA followed by post-hoc Bonferroni tests; ***p < 0.001 versus the 85As2 + DW-treated group (B). Differences before (Pre) and after (Post) administration of RKT or DW were evaluated using paired *t*-tests; ## *p* < 0.01, ###*p* < 0.001 versus the corresponding group before administration (each baseline was set as 0) (C, D). Differences between groups were evaluated using one-way ANOVA followed by post-hoc Dunnett’s multiple comparison tests; **p < 0.01 versus the 85As2 + DW-treated group (D). RKT: rikkunshito; DW: distilled water; n.s.: not significant.

CC clearly developed at day 14, i.e., 2 weeks after implantation of 85As2 cells (S1 Fig); after this time, the rats were administered RKT or DW for 7 days. In the 85As2-implanted group, progressive tumor growth was observed during the experiment, and RKT did not affect tumor volume (Fig 6A). Two-way repeated measures ANOVA of tumor volume revealed significant effects of time [F (3, 81) = 149.8, *p* < 0.001], but not treatment [F (1, 81) = 0.001769, n.s.] or treatment × time interaction [F (3, 81) = 0.1338, n.s.]. The body weights of rats in the Control + DW group continued to increase as compared to those in both the 85As2 + DW and 85As2 + RKT groups during the experiment (Fig 6B). Two-way repeated measures ANOVA of body weights in all groups revealed significant effects of treatment (group) [F (2, 294) = 25.97, *p* < 0.001], time [F (14, 294) = 15.96, *p* < 0.0001] and treatment × time interaction [F (14, 294) = 21.24, *p* < 0.0001]. The body weights of rats in the Control + DW group were significantly higher than those in the 85As2 + DW group (two-way repeated measures ANOVA followed by Bonferroni post-hoc tests, days 16–21: *p* < 0.001; Fig 6B), reaching 105.6% ± 0.5% on day 21, and were significantly increased as compared to those before administration [paired *t*-tests, Pre (day 14) versus Post (day 21): *p* < 0.001; Fig 6C)]. In contrast, the body weights of rats in the 85As2 + DW group gradually decreased, falling to 97.8% ± 0.7% on day 21, showing significant decreases as compared to those before administration (paired *t*-tests, Pre versus Post: *p* < 0.01; Fig 6C). In contrast, the body weights of rats in the 85As2 + RKT group were not changed as compared to those before administration, measuring 99.6% ± 0.8% on day 21 (Figs 6B and 6C). Thus, administration of RKT alleviated body weight loss.

In both the 85As2 + DW group and the 85As2 + RKT group, daily food intake before administration was significantly decreased 2 weeks after implantation as compared to that in the Control + DW group (S1 Fig). Subsequently, in the 85As2 + RKT group, daily average food intake after administration from day 16 to 21 significantly increased as compared to that before administration (paired *t*-tests, *p* < 0.001; Fig 6D), whereas those of the Control + DW and 85As2 + DW groups showed only very slight increases (daily food intake gain: Control + DW group, 0.20 ± 0.34 g; 85As2 + DW group, 0.22 ± 0.52 g; 85As2 + RKT group, 2.38 ± 0.55 g; Fig 6D). One-way ANOVA of average daily food intake gain in all groups revealed significant effects [F (2, 42) = 6.839, *p* < 0.01]. The differences between the 85As2 + DW and 85As2 + RKT groups were significant (one-way ANOVA followed by post-hoc Dunnett’s multiple comparison tests, *p* < 0.01; Fig 6D).

In 85As2 + DW group, muscle (greater pectoral, gastrocnemius, soleus, and tibialis anterior), adipose tissue (epididymal, perirenal, and mesentery fat), liver and spleen weights substantially decreased as compared to those in Control + DW group (Table 2). These reduction caused by cachexia appeared to be greater than those in the 85As2 group in Table 1 because tumor growth and cachexia progressed much more during until 21 days from 14 days. RKT treatment significantly increased greater pectoral muscle and total muscle weights as compared to those in the 85As2 + DW group (Aspin–Welch’s *t*-test, *p* < 0.05; Table 2).

**Table 2:** Body, tumor, muscle, fat, and organ weights in 85As2-induced CC rats with or without RKT treatment.

		Control +DW	85As2+DW	85As2+RKT
Tumor weight (T.W.)		0.00 ± 0.00	7.74 ± 0.47	7.76 ± 0.72
Tumor volume		0.00 ± 0.00	11.03 ± 1.03	12.35 ± 1.37
Body weight (B.W.)		252.90 ± 4.00	194.71 ± 3.40***	198.48 ± 3.19***
B.W.–T.W.		252.90 ± 4.00	186.97 ± 3.36***	190.72 ± 2.97***
B.W. (pre-inoculation)		190.78 ± 3.11	190.81 ± 2.78	189.99 ± 2.94
% Change (post/pre)		132.62 ± 1.07	98.02 ± 1.32***	100.44 ± 1.15***
Muscle weights				
	Greater pectoral	2.31 ± 0.07	1.60 ± 0.06***	1.84 ± 0.08***, #
	Gastrocnemius	1.33 ± 0.02	1.05 ± 0.02***	1.08 ± 0.02***
	Tibialis anterior	0.50 ± 0.01	0.40 ± 0.01***	0.42 ± 0.01***
	Soleus	0.08 ± 0.00	0.07 ± 0.00***	0.07 ± 0.00***
	Total muscle	6.14 ± 0.09	4.62 ± 0.12***	5.00 ± 0.11***, #
Fat weights				
	Epididymis	3.36 ± 0.17	1.50 ± 0.08***	1.50 ± 0.06***
	Perirenal	2.73 ± 0.16	0.58 ± 0.08***	0.72 ± 0.06***
	Mesentery	2.16 ± 0.10	0.51 ± 0.08***	0.68 ± 0.07***
	Total fat	8.25 ± 0.40	2.58 ± 0.24***	2.90 ± 0.18***
Organ weights				
	Liver	10.27 ± 0.29	6.98 ± 0.22***	7.43 ± 0.18***
	Spleen	0.56 ± 0.02	0.47 ± 0.02***	0.46 ± 0.01***

Rats were s.c. implanted with either 85As2 cells (1 × 10^7^ cells/site) or saline in both flanks. Data are expressed as the mean ± SEM of 10–12 rats. All weight data are expressed in grams. Tumor volume was estimated using the following equation: tumor volume (cm^3^) = major axis (cm) × minor axis (cm) × minor axis (cm) × 1/2. Tumor weight and volume are expressed as the total for both sites. Body and tissue weights were measured on day 21. % Change (post/pre) was expressed as follows: % change = tumor-free body weight (B.W.–T.W.)/body weight of pre-inoculation with 85As2 cells or saline on day 0 × 100. Values for bilateral muscle tissues represent the mean of those for the 2 unilateral tissues. Differences between groups were evaluated using the Aspin–Welch *t*-test.

*p < 0.05,

**p < 0.01,

***p < 0.001 vs. the Control + DW group,

^#^*p* < 0.05 vs. the 85As2 + DW group.

**Fig 7:**
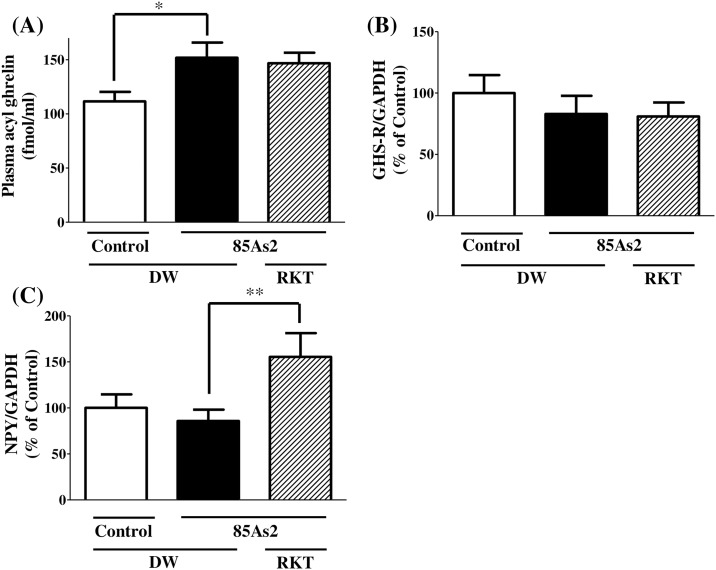
Effects of RKT on plasma acyl ghrelin levels and hypothalamic *GHS-R* and *NPY* mRNAs in 85As2-induced CC rats. (A) Plasma acyl ghrelin levels and (B) *GHS-R* and (C) *NPY* mRNAs in the hypothalamus in control and 85As2-induced CC rats with or without RKT administration 3 weeks after implantation. Rats were implanted subcutaneously with 85As2 cells in both flanks (1 × 10^7^ cells/site). Two weeks after implantation, RKT (1 g/kg/day) or DW was administered orally twice a day for 7 days. Rats inoculated with saline served as a non-tumor-bearing control group and were administered DW. Plasma or hypothalamus samples were collected after administration of RKT for 7 days. Each column represents the mean ± SEM of 10–12 rats. Differences between groups were evaluated using one-way ANOVA followed by Dunnett’s multiple comparison tests. *p < 0.05, **p < 0.01 versus the 85As2 + DW-treated group. RKT: rikkunshito; DW: distilled water; GHS-R: growth hormone secretagogue receptor.

Plasma ghrelin levels were significantly elevated in 85As2-bearing CC rats compared to those in non-tumor-bearing rats (one-way ANOVA, F (2, 28) = 4.167, *p* < 0.05, post-hoc Dunnett’s multiple-comparison tests, *p* < 0.05; Fig 7A). In contrast, RKT administration for 7 days did not affect the elevated plasma ghrelin levels in 85As2-bearing CC rats. Hypothalamic *GHS-R* gene expression levels did not change in 85As2-bearing rats as compared to those in non-tumor-bearing rats (Fig 7B). In addition, RKT treatment also failed to alter the expression of this gene in 85As2 tumor-bearing rats. Hypothalamic *NPY* gene expression did not change in 85As2 tumor-bearing rats as compared to that in non-tumor-bearing rats. However, RKT treatment significantly increased hypothalamic *NPY* gene expression in 85As2 tumor-bearing rats (one-way ANOVA, F(2, 30) = 3.784, *p* < 0.05, post hoc Dunnett’s multiple-comparison tests, *p* < 0.01; Fig 7C). In a preliminary study, hypothalamic feeding-regulating peptide mRNA levels were evaluated by in situ hybridization. When compared with 85As2 CC rats, RKT significantly reduced the expression of *TRH* in the PVN and increased the expression of *NPY* in the ARC; however, this latter difference was not significant (S2 Fig).

### Preventive effects of long-term feeding of RKT diet on anorexia in the 85As2-induced CC model

**Fig 8:**
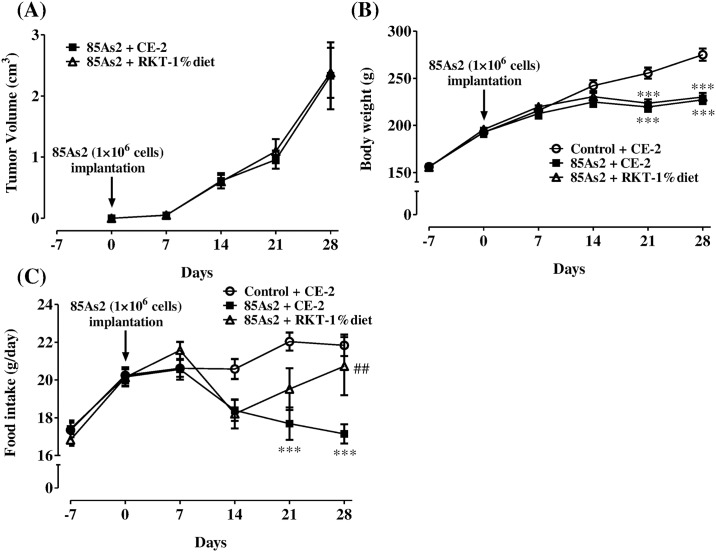
Effects of long-term feeding of RKT (1% diet) from before tumor implantation on tumor growth or anorexia in the 85As2-induced CC model. Changes in (A) tumor volume, (B) body weight, and (C) average daily food intake during the experiment. Rats were implanted subcutaneously with 85As2 cells in both flanks (1 × 10^6^ cells/each site) on day 0. RKT (CE-2 diet containing 1% RKT) or CE-2 was fed for 35 days from day –7. Rats inoculated with saline served as a non-tumor-bearing control group and were fed similarly for 35 days. Each data point represents the mean ± SEM of 9–11 rats. Differences between groups in the time course of body weight and food intake were evaluated using two-way repeated measures ANOVA followed by Bonferroni post-hoc test; ***p < 0.001 versus the corresponding Control + CE-2 group, ##*p* < 0.01 versus the corresponding 85As2 + CE-2 group. RKT: rikkunshito.

For evaluation of the long-term effects of RKT, we used a model in which CC gradually developed. That is, rats were implanted with one-tenth the number of 85As2 cells (1 × 10^6^ cells/site) used in the conventional CC model. Tumors grew slowly, and cachexia symptoms, such as body weight loss and anorexia, were not observed at 14 days after tumor implantation, unlike in rats inoculated with 1 × 10^7^ cells/site (Fig 8A–8C). Two-way repeated measures ANOVA of body weight in all groups revealed significant effects of treatment (group) [F (2,140) = 5.001, *p* < 0.05], time [F (5, 140) = 706.7, *p* < 0.0001] and treatment × time interaction [F (10, 140) = 31.73, *p* < 0.0001]. Furthermore, two-way repeated measures ANOVA of food intake in all groups revealed significant effects of treatment [F (2,140) = 3.714, *p* < 0.05], time [F (5, 140) = 17.02, *p* < 0.0001] and treatment × time interaction [F (10, 140) = 5.146, *p* < 0.0001]. In the tumor-bearing control (85As2 + CE2) group, body weight and daily food intake were significantly decreased after 21 days (two-way repeated measures ANOVA followed by Bonferroni post-hoc tests, *p* < 0.001) as compared to those in the non-tumor-bearing (control + CE-2) group, and these differences appeared to be greater at 28 days (Bonferroni tests, *p* < 0.001; Fig 8B and 8C). In contrast, feeding of the RKT-1% diet beginning before tumor implantation significantly ameliorated reduced food intake on day 28 in 85As2-induced CC rats (post-hoc Bonferroni tests, *p* < 0.01; Fig 8C), whereas the treatment did not affect tumor growth or body weight loss in CC rats (Fig 8A and 8B).

## Discussion

In the present study, we found that our cancer anorexia-cachexia rat model, induced by human gastric cancer-derived 85As2 cells, developed ghrelin resistance. This finding suggested that ghrelin resistance may be involved in the development of anorexia in our CC model in the presence of elevated plasma ghrelin. RKT, which enhances ghrelin signaling through GHS-R, partly alleviated ghrelin resistance and ameliorated cachexia symptoms, such as anorexia and body weight loss, without affecting elevated plasma ghrelin in the model. These results suggested that the mechanisms through which RKT ameliorated cancer anorexia-cachexia symptoms in this model may involve alleviation of ghrelin resistance, possibly through enhancement of ghrelin signaling.

We showed that 85As2-implanted rats clearly exhibited reproducible cachexia symptoms, including anorexia, body weight loss, and reduced muscle and adipose tissues, up to 14 days after implantation. Body weights of pair-fed rats were reduced to levels similar to those in 85As2-induced cachexia rats, in accordance with decreased food consumption. However, when the tumor weight was subtracted, the body weights of 85As2-induced cachexia rats were lower than those of pair-fed rats, supported by greater decreases in muscle and adipose tissues in the 85As2-induced CC rats. The differences in body weight losses between pair-fed and CC rats were likely caused by factors other than decreased food consumption. We previously demonstrated that energy expenditure was enhanced in 85As2-induced CC rats when compared to that in control rats [10]. Therefore, enhanced energy expenditure in CC rats may have exacerbated body weight loss, including decreases in adipose and muscle tissues. On the other hand, with respect to orexigenic peptides (*NPY* and *AgRP* mRNAs in the ARC, *ORX* and *MCH* mRNAs in the LHA) or anorexigenic peptides (*POMC* and *CART* mRNAs in the ARC, *CRH* and *TRH* mRNAs in the PVN) in the hypothalamus, the mRNA levels mostly exhibited similar variations in 85As2-induced CC and pair-fed rats. Therefore, the changes in these hypothalamic peptide mRNAs in 85As2-induced CC rats appeared to be involved in decreased food consumption. However, despite similar food consumption, daily water intake was markedly reduced in 85As2-cachectic rats, whereas the reduction in pair-fed rats was minor. This finding suggested that dysfunction in the central nervous system controlling body fluid, such as water intake when perceiving the dehydration state, may emerge in cachectic rats; however, further studies are needed.

Interestingly, rats with 85As2-induced CC 2 weeks after implantation showed poor sensitivity to exogenous ghrelin, suggesting that ghrelin resistance developed under these pathological conditions. Ghrelin-induced appetite stimulation mainly occurred through activation of NPY/AgRP neurons in the ARC of the hypothalamus, a major site for appetite regulation in the central nervous system. Circulating ghrelin, secreted by the stomach, is transported across the blood brain barrier and binds to GHS-R located in NPY/AgRP neurons, resulting in signal transmission to the hypothalamus. The activation of GHS-R in NPY/AgRP neurons of the hypothalamus stimulates appetite. Additionally, peripheral ghrelin binds to GHS-R located in the gastric afferent vagus nerve and transmits a ghrelin signal to NPY/AgRP neurons in the hypothalamus [37]. Thus, GHS-R activation by ghrelin in NPY/AgRP neurons of the hypothalamus through these peripheral pathways stimulates appetite. In 85As2-induced CC rats, plasma ghrelin levels were significantly elevated. This finding has been also reported for patients with CC [20, 21], supporting our results. Because fasting and a negative energy balance increase plasma ghrelin [38], the elevated ghrelin levels in our CC rat model could be a compensatory effect in response to the negative energy balance. However, food intake was continuously decreased in the cachectic rats, suggesting that endogenous ghrelin-induced appetite stimulation was not physiologically produced. Subsequently, to elucidate the mechanisms through which administration of exogenous ghrelin to cachectic rats did not promote sufficient food intake, we evaluated *GHS-R* gene expression in the hypothalamus in 85As2-induced cachectic rats and found that *GHS-R* expression in cachectic rats was comparable to that in non-tumor-bearing rats. Therefore, it is unlikely that the development of ghrelin resistance in cachectic rats was caused by attenuated response to ghrelin via the decrease in GHS-R expression.

We previously found that cultured 85As2 cells produce LIF, which is an inflammatory cytokine and cachectic factor [7]. The LIF receptor is localized in anorexigenic POMC/CART neurons [39]. Consistent with the results in 85As2 cell cultures, plasma LIF levels increased in 85As2-bearing rats with the development of cachexia symptoms, and surgical removal of the tumor not only abolished cachexia symptoms but also reduced plasma LIF levels to below the detection limit, demonstrating that LIF contributes to cachexia symptoms in the 85As2-induced CC model [10]. Thus, LIF might contribute to the observed ghrelin resistance in 85As2-induced CC; however, further studies are required to clarify the association.

RKT is widely used for the treatment of gastrointestinal motor dysfunction symptoms [40]. RKT has been shown to ameliorate anorexia by increasing ghrelin secretion. Takeda et al. reported that RKT increases ghrelin secretion via serotonin 2b/2c receptor antagonism and ameliorates anorexia induced by the anticancer drug cisplatin [25]. Additionally, Matsumura et al. reported that RKT increases plasma ghrelin concentrations in mice and humans [26], and Arai et al. reported that RKT alleviates upper gastrointestinal symptoms in patients with functional dyspepsia accompanied by increased plasma ghrelin [28]. More recently, RKT has been shown to potentiate ghrelin signaling by enhancing GHS-R activity [29, 30]. In the present study, we investigated whether RKT could directly enhance ghrelin signaling by using the CellKey system, which is specifically tailored to G protein-coupled receptor detection and produces response profiles that are consistent across a wide range of assay conditions, with the capacity for distinguishing signals between Gs, Gi/o, and Gq subtypes of Gα protein [41, 42]. In addition, this system enables unlabeled, whole-cell, real-time, and high-throughput assays and can be used to measure the integrated response of whole cells to receptor activation by measuring impedance in cells [41]. That is, impedance changes in cells are dependent on the type of G protein, coupled with a specific receptor. GHS-R is a Gq protein-coupled receptor; therefore, ghrelin stimulation in GHS-R-expressing HEK293A cells induced Gq-specific impedance changes in a concentration-dependent manner. RKT further enhanced ghrelin-induced impedance changes, suggesting that RKT directly enhanced ghrelin signaling following the binding of ghrelin to GHS-R. This result was consistent with previous reports using GHS-R-expressing cells [29, 30].

In the present study, we found that ghrelin resistance developed in 85As2-induced CC rats at 2 weeks after implantation; therefore, we examined whether RKT, which enhances ghrelin signaling, alleviated ghrelin resistance. Interestingly, in 85As2-bearing CC rats (85As2 + DW), at 3 weeks after implantation, i.p. injection of ghrelin did not significantly increase 1-h food intake, suggesting that ghrelin resistance development accelerated between 2 and 3 weeks after implantation. RKT administration for 7 days partly ameliorated the poor sensitivity to exogenous ghrelin in cachectic rats at 3 weeks after implantation, suggesting that RKT alleviated ghrelin resistance. RKT alleviated the cachexia-induced decrease in food intake both at 1 h after ghrelin injection and at 22 h after ghrelin or vehicle injection. Thus, RKT is likely to enhance the sensitivity to endogenous ghrelin by potentiating ghrelin signaling and to increase 22-h food intake, as the ghrelin injection-induced increase in food intake lasted for only 1–2 h. This speculation was supported by results demonstrating that daily average food intake in individual rats was significantly increased by RKT administration. Furthermore, the losses in both body weight and muscle mass were also attenuated after RKT administration, demonstrating that RKT ameliorated anorexia-cachexia symptoms in the 85As2-induced CC model, as reported previously [10]. In addition to the effects of treatment with RKT on emerging cachexia, we found that RKT pretreatment beginning before tumor implantation prevented anorexia in the 85As2-induced CC model. However, amelioration of CC by RKT was not due to augmentation of ghrelin secretion because RKT did not affect plasma ghrelin levels in CC rats. Additionally, RKT did not affect tumor growth. RKT also did not alter plasma LIF levels, as reported previously [10]. These findings indicated that the anticachectic effects of RKT were not related to tumor regression or LIF levels.

Subsequently, we found that RKT did not affect hypothalamic *GHS-R* gene expression in 85As2-induced CC rats, suggesting that the alleviation of ghrelin resistance by RKT was not due to the increase in *GHS-R* expression. Yakabi et al. previously reported that RKT significantly reversed the reduction in *GHS-R* gene expression induced by cisplatin, whereas RKT did not affect *GHS-R* expression in normal rats [43]. This result suggested that the acute decrease in food intake induced by cisplatin was involved in the reduction in *GHS-R* gene expression. In contrast, in the present study, ghrelin resistance in cachectic rats was shown to be involved in the attenuated response following the binding of ghrelin to GHS-R because *GHS-R* gene expression in cachectic rats was comparable to that in non-tumor-bearing rats. Therefore, our findings suggest that alleviation of ghrelin resistance and amelioration of anorexia in cachectic rats treated with RKT occurs via the enhancement of ghrelin signaling following GHS-R binding rather than through increased GHS-R expression. Improvement of appetite by ghrelin is involved in the regulation of the orexigenic peptide NPY. Therefore, we investigated the effects of RKT-dependent enhancement of ghrelin signaling on hypothalamic *NPY* expression. We found that RKT increased *NPY* expression in the hypothalamus of 85As2-induced cachectic rats. In an *in vitro* study, RKT also enhanced the ghrelin-induced increase in intracellular Ca^2+^ in NPY neurons, which express GHS-R, isolated from the ARC [29]. These findings suggest that the activation of NPY neurons via enhancement of ghrelin signaling by RKT may contribute to the alleviation of ghrelin resistance and the amelioration of anorexia in cachectic rats.

Subsequently, we examined the effects of RKT on hypothalamic orexigenic/anorexigenic peptides in 85As2-induced cachectic rats. The expression of *NPY* mRNA in the ARC was higher in cachectic rats treated with RKT than in those not treated with RKT, although the difference was not significant. Our previous study demonstrated that RKT ameliorated cisplatin-induced anorexia in rats and reversed the cisplatin-induced decrease in hypothalamic *NPY* gene expression in the ARC [34]. In the present preliminary study, however, we did not observe these effects of RKT. In the hypothalamus, both the ARC and PVN have been shown to play important roles in the enhancement of food intake by ghrelin administration [44]. The preliminary study also demonstrated that RKT significantly decreased *TRH* gene expression in the PVN as compared to that in 85As2-induced CC rats. TRH is an anorexigenic peptide, and central injection of TRH reduces food intake in rats [45]. Additionally, TRH neurons in the PVN are densely innervated from NPY-containing axon terminals of ARC [46–48]. Moreover, intracerebroventricular administration of NPY decreases *TRH* gene expression in the PVN [49]. Taken together, this reduction in *TRH* gene expression in the PVN in cachectic rats by RKT may be partly involved in the amelioration of anorexia-cachexia by RKT. However, these mechanisms are still not fully elucidated, and further studies are required to clarify the association of the anticachectic effects of RKT and variations in orexigenic/anorexigenic peptides modified by RKT in the hypothalamic region, including ARC, PVN, and LHA. In contrast, RKT directly affects the stomach and is known to facilitate gastric emptying, gastric adaptive relaxation, and upper gastrointestinal motility [50–52]. These effects may synergistically ameliorate cachexia-anorexia.

In conclusion, we demonstrated that our cancer anorexia-cachexia rat model, induced by human gastric cancer-derived 85As2 cells, exhibited ghrelin resistance, possibly contributing to anorexia and body weight loss. The mechanism through which RKT ameliorated cancer anorexia-cachexia symptoms may involve alleviation of ghrelin resistance via enhancement of ghrelin signaling. These findings suggest that RKT may be a promising agent for the treatment of patients with CC who show high levels of circulating ghrelin.

## Supporting information

S1 Fig Changes in (a) body weight and (b) food intake before RKT administration in the 85As2-induced cancer cachexia model. Rats were implanted s.c. with 85As2 cells in both flanks (1 × 10^7^ cells/site) on day 0. Rats inoculated with saline served as a non-tumor-bearing control group. Each data point or column represents the mean ± SEM of 14–16 rats. Differences between groups in body weight and food intake over time were evaluated using two-way repeated measures ANOVA followed by Bonferroni post-hoc tests. **p < 0.01, ***p < 0.001 versus the corresponding Control + DW group. RKT: rikkunshito; DW: distilled water.
(TIFF)

S2 Fig Effects of RKT on hypothalamic orexigenic/anorexigenic peptides in 85As2-induced CC rats. (a) Expression of mRNAs encoding neuropeptide Y (*NPY*), agouti-related protein (*AgRP*), proopiomelanocortin (*POMC*), and cocaine- and amphetamine-regulated transcript (*CART*) in the arcuate nucleus (ARC); corticotropin-releasing hormone (*CRH*) and thyrotropin-releasing hormone (*TRH*) in the paraventricular nucleus (PVN); and orexin (*ORX*) and melanin-concentrating hormone (*MCH*) in the lateral hypothalamic area (LHA) was measured by *in situ* hybridization. (b) *GHS-R* and (c) *NPY* mRNAs in the hypothalamus in control and 85As2-induced CC rats with or without RKT administration 3 weeks after implantation. The rats were implanted s.c. with 85As2 cells in both flanks (1 × 10^7^ cells/site). Two weeks after implantation, RKT (1 g/kg/day) or DW was administered orally twice a day for 7 days. Rats inoculated with saline served as a non-tumor-bearing control group and were administered DW. Hypothalamus samples were collected after administration of RKT for 7 days. Hypothalamic mRNAs encoding orexigenic/anorexigenic peptides in each brain region (ARC, PVN, or LHA) were measured by *in situ* hybridization. Each column represents the mean ± SEM of six rats. Differences between groups were evaluated using one-way ANOVA followed by post-hoc Bonferroni tests; #*p* < 0.05 versus the 85As2 + DW group. RKT: rikkunshito; DW: distilled water.
(TIFF)
