# Supplementary figures and images for: Development of ghrelin resistance in a cancer cachexia rat model using human gastric cancer-derived 85As2 cells and the palliative effects of the Kampo medicine rikkunshito on the model
Source: PLoS One. 2017 Mar 1;12(3):e0173113. doi: 10.1371/journal.pone.0173113 (PMC5332064; doi:10.1371/journal.pone.0173113)

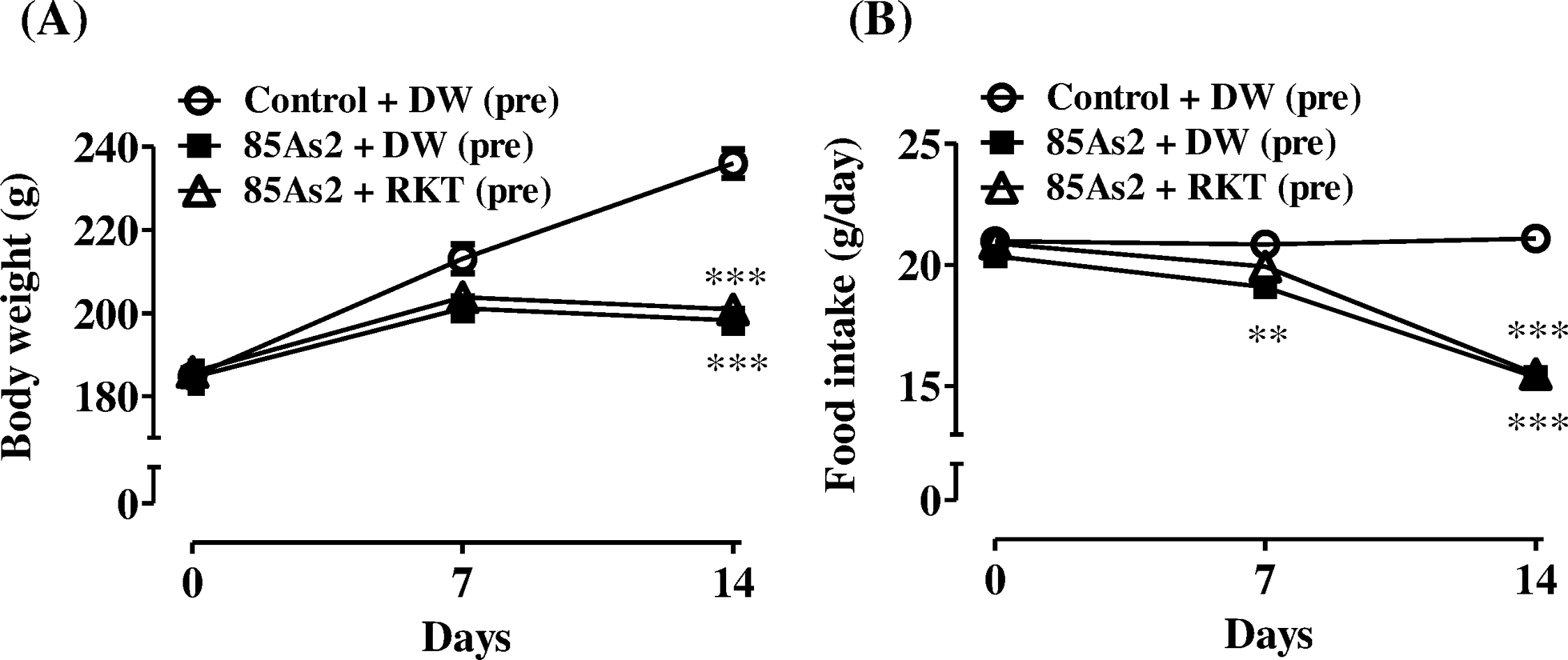

Supplement: S1 Fig — Rats were implanted s.c. with 85As2 cells in both flanks (1 × 10^7^ cells/site) on day 0. Rats inoculated with saline served as a non-tumor-bearing control group. Each data point or column represents the mean ± SEM of 14–16 rats. Differences between groups in body weight and food intake over time were evaluated using two-way repeated measures ANOVA followed by Bonferroni post-hoc tests. **p < 0.01, ***p < 0.001 versus the corresponding Control + DW group. RKT: rikkunshito; DW: distilled water. (TIFF) [file pone.0173113.s001.tiff]

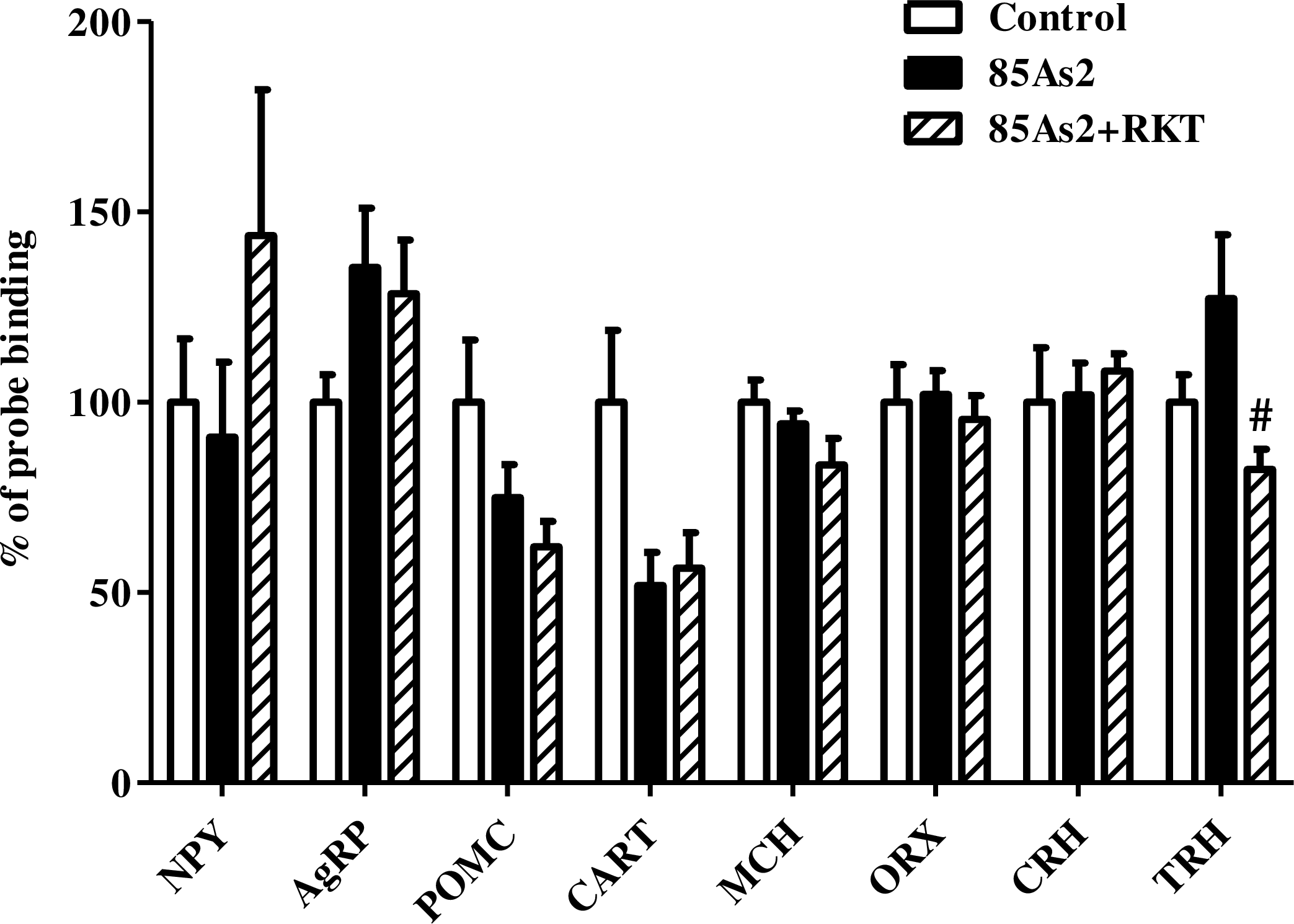

Supplement: S2 Fig — (a) Expression of mRNAs encoding neuropeptide Y (NPY), agouti-related protein (AgRP), proopiomelanocortin (POMC), and cocaine- and amphetamine-regulated transcript (CART) in the arcuate nucleus (ARC); corticotropin-releasing hormone (CRH) and thyrotropin-releasing hormone (TRH) in the paraventricular nucleus (PVN); and orexin (ORX) and melanin-concentrating hormone (MCH) in the lateral hypothalamic area (LHA) was measured by in situ hybridization. (b) GHS-R and (c) NPY mRNAs in the hypothalamus in control and 85As2-induced CC rats with or without RKT administration 3 weeks after implantation. The rats were implanted s.c. with 85As2 cells in both flanks (1 × 10^7^ cells/site). Two weeks after implantation, RKT (1 g/kg/day) or DW was administered orally twice a day for 7 days. Rats inoculated with saline served as a non-tumor-bearing control group and were administered DW. Hypothalamus samples were collected after administration of RKT for 7 days. Hypothalamic mRNAs encoding orexigenic/anorexigenic peptides in each brain region (ARC, PVN, or LHA) were measured by in situ hybridization. Each column represents the mean ± SEM of six rats. Differences between groups were evaluated using one-way ANOVA followed by post-hoc Bonferroni tests; #p < 0.05 versus the 85As2 + DW group. RKT: rikkunshito; DW: distilled water. (TIFF) [file pone.0173113.s002.tiff]
